# Clinical Assessment of Nifedipine-Induced Gingival Overgrowth in a Group of Brazilian Patients

**DOI:** 10.5402/2011/102047

**Published:** 2011-06-29

**Authors:** Cliciane Portela Sousa, Claudia Maria Navarro, Maria Regina Sposto

**Affiliations:** Department of Diagnosis and Oral Surgery, Dental School, UNESP, 14801-903 Araraquara, SP, Brazil

## Abstract

Although it has been established that nifedipine is associated with gingival overgrowth (GO), there is little information on the prevalence and severity of this condition in the Brazilian population. The aim of this study was to assess the occurrence of nifedipine-induced GO in Brazilian patients and the risk factors associated using a Clinical Index for Drug Induced Gingival Overgrowth (Clinical Index DIGO). The study was carried out on 35 patients under treatment with nifedipine (test group) and 35 patients without treatment (control group). Variables such as demographic (age, gender), pharmacological (dose, time of use), periodontal (plaque index, gingival index, probing depth, clinical insertion level, and bleeding on probing), and GO were assessed. Statistical analysis showed no association between GO and demographic or pharmacological variables. However, there was an association between GO and periodontal variables, except for plaque index. According to our study, the Clinical Index DIGO can be used as a parameter to evaluate GO. Therefore, we conclude that the presence of gingival inflammation was the main risk factor for the occurrence of nifedipine-induced GO.

## 1. Introduction

Drug-induced gingival overgrowth (DIGO) is a histomorphological alteration due to the side effects of a medication on the extracellular matrix [1]. Several drugs induce gingival overgrowth, but phenytoin, cyclosporine, and nifedipine produce significant alterations in terms of prevalence and severity of gingival overgrowth [2].

Nifedipine is a specific calcium antagonist which inhibits calcium influx directly from the cells of cardiac muscle and has a vasodilatory action that causes reduced arterial pressure [3]. Among calcium antagonists, it is the drug most commonly related to DIGO [4], whose prevalence ranges from 20% to 83% [5, 6].

According to Seymour et al. [7], the hypothesis for the etiology of DIGO is multifactorial. Analysis of variables such as demographic (patient's age and gender), pharmacological (dose, time of use, serum, and salivary concentration of the drug) and periodontal (plaque index and gingival inflammation), in addition to genetic factors and association of medications, have been identified as risk factors for this condition [7, 8].

The correlation between demographic and pharmacological variables to the extent and severity of GO has been studied, aiming at the identification of risk situations for the patients under nifedipine treatment [8–10]. Concerning the periodontal variables, some studies have suggested a consensus about the fact that bacterial plaque and gingival inflammation are risk factors strongly associated with nifedipine-induced GO [5, 7, 11]. 

Most of the indexes used and reported in the literature for the assessment of DIGO are complicated to use, many of them require the preparation of plaster casts and many measurements and procedures that impair their routine use [5, 7, 11].

Considering the lack of studies on the prevalence and severity of DIGO related to the use of nifedipine in the Brazilian population, this study was proposed. Thus, the objective was to assess nifedipine DIGO in a Brazilian group of patients and evaluate the possible association with demographic, pharmacological, and periodontal variables using the Clinical Index for Drug-Induced Gingival Overgrowth (DIGO) proposed by Inglés et al. [11].

## 2. Material and Methods

### 2.1. Patient Selection

The protocol for patient care was approved by the Research Ethics Committee of the Dental School of Araraquara, UNESP, São Paulo, Brazil. Two groups of patients, test and control, were used. The test group was selected at the Health Stations of the Municipality of Araraquara, São Paulo, Brazil, among patients with cardiovascular disease who were under periodical medical control and who had been taking nifedipine for at least 6 months under medical monitorization. The control group was selected among patients who sought the Dental School of Araraquara for dental treatment. 

The following inclusion criteria were used for the test and control groups: no periodontal treatment for the preceding 6 months, no use of orthodontic braces or dentures, absence of defective restorations, and the presence of at least 6 to 12 teeth from the anterior region. Exclusion criteria were *diabetes mellitus*, blood dyscrasia, hormonal changes, pregnancy, oral breathing, smoking, patients taking systemic antibiotics, or anti-inflammatory drugs (steroidal and non-steroidal), and patients taking contraceptive medication or any other drug inducing gingival overgrowth.

### 2.2. Clinical Examination

During clinical examination, a single examiner (MRS) performed the anamnesis and a chart was filled out with the identification for Group Test or Control, patient age, gender, and pharmacological data (dose and time of use of nifedipine). The gingival areas involved in the study were photo-documented. 

### 2.3. Periodontal Examination

Gingival Overgrowth (GO) was assessed in the upper teeth by the method of Inglés et al. [11]. This method consists of clinical evaluation of the vestibular and lingual papillae, which were scored from 0 to 4 according to the Clinical Index DIGO [11]. A single examiner (CPS) blind for identification of patients, trained and calibrated for the Kappa agreement test, performed the periodontal examination, which consisted of the measurement of the following variables: plaque index (PI) [12], gingival index (GI) [13], probing depth (PD), bleeding on probing (PB), and clinical insertion level (CIL). 

### 2.4. Criteria for the Analysis of Periodontal Variables and Gingival Overgrowth

The data collected during the periodontal examination were divided into groups for statistical analysis. The variables PI and GI were divided into groups according to the absence or presence of visible plaque and according to marginal probing bleeding, respectively, with scores of 0 (presence) and 1 (absence). PB variable was divided into groups according to the absence or presence of bleeding on probing, with scores varying from 0 (presence) to 1 (absence). The PD and CIL variables were divided into groups according to intervals of sites with PD and CIL < 3 mm, from 3 to 4 mm, and ≥5 mm. Finally, GO was divided into groups according to absence (score < 2) and presence (score ≥ 2).

### 2.5. Statistical Analysis

The values obtained for the test and control groups concerning the periodontal variables (PI, GI, PD, PB, and CIL) and GO were submitted to the *Z* test at the 5% level of significance (*P* < .05) for the comparison of proportions of relative frequencies [14]. The Spearman correlation test using the BioEstat 2.0 software [15] was used to calculate the correlations between GO and the demographic, pharmacological, and periodontal variables. 

## 3. Results

The patient's data are presented in Table 1, which shows a higher prevalence of males in the test group, with a mean age of 69.5 years and a mean daily dose of 40 mg for a mean period of 13 years (156 months) of nifedipine use.

With the use of the Clinical Index DIGO [11], GO was observed in 68% of the patients in the test group and in 23% of the patients in the control group. Application of the *Z* test demonstrated a significant difference in GO between groups at the 5% level of significance (*P* < .05). The values of the *Z* test for the comparison of the proportions of GO and of the periodontal variables between groups are listed in Table 2. The *Z* test showed a no significant difference for PI, GI, PD, and PB between the test and control groups. However, for CIL the *Z* test showed a statistically significant difference for the intervals with CIL < 3 mm and CIL from 3 to 4 mm. 

**Figure 1:**
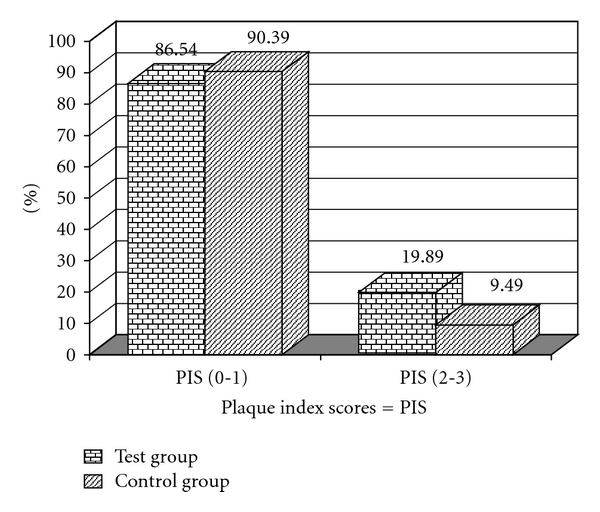
Percentages of sites with dichotomized Plaque Index Scores (0-1) and (2-3) for the test (T) and control (C) groups.

**Figure 2:**
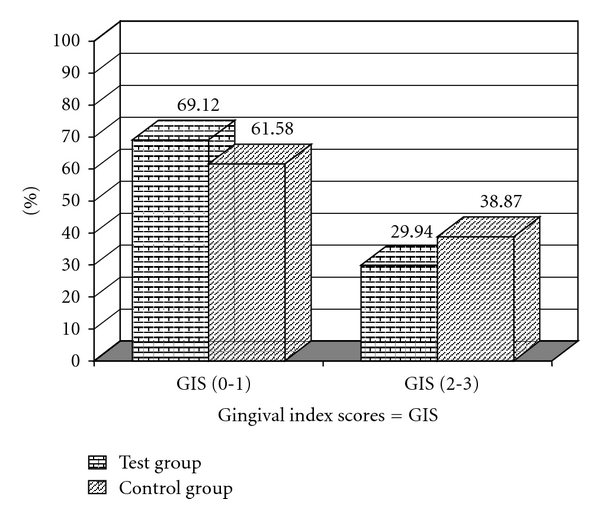
Percentages of sites with dichotomized Gingival Index Scores (0-1) and (2-3) for the test (T) and control (C) groups.

**Figure 3:**
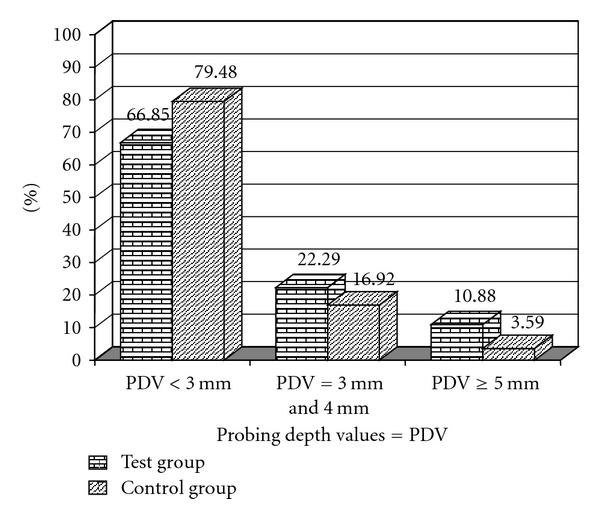
Percentages of sites with Probing Depth Values < 3 mm, from 3 to 4 mm, and ≥5 mm for the test (T) and control (C) groups.

**Figure 4:**
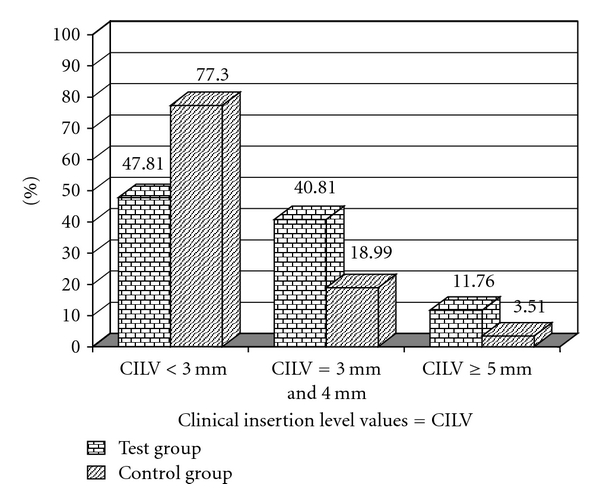
Percentages of sites with Clinical Insertion Level Values < 3 mm, from 3 to 4 mm, and ≥5 mm for the test (T) and control (C) groups.

In Figures 1 and 2 we can observe a higher frequency for the scores 2 and 3 for PI in the test group and for GI in the control group. Figures 3 and 4 illustrate the mean percentages of PD and CIL, demonstrating higher PD and CIL frequencies in the interval of 3 to 4 mm for both groups and in the PD ≥ 5 mm and CIL ≥ 5 mm interval for the test group.

The demographic variables (age and gender) and the pharmacological variables (dose and time of use of nifedipine) did not show correlation with GO (Table 3). A correlation between degree of severity of GO and CIL < 3 mm and CIL ≥ 5 mm was detected only for the test group (Table 4). The periodontal variable PI was correlated with the degree of severity of GO only in the control group. There was a positive correlation between GI, PD, and PB and the degree of GO severity for both groups. 

## 4. Discussion

Since the first report of drug-related GO by Kimbal [16] in 1939, several studies have been conducted in an attempt to understand the factors that act on this process. Today, more than 20 drugs are known to induce GO [2].

Few studies are currently available in the literature about the influence of nifedipine in gingival manifestations. In the present study, the mean age of patients in the test group was 69.5 years and the age variable did not show a correlation with GO, in agreement with other studies [6, 9, 10, 17]. However, according to Thomason et al. [18] and James et al. [19], younger patients show a higher prevalence of GO when the association of nifedipine and cyclosporine treatment was identified. Maybe this is an effect of drug association and not only related to the age of the patients.

In our group of patients, despite the larger number of male patients in the test group than in the control group (22 : 6), GO was more prevalent among females in both groups (63.63% for the test group and 76.92% for the control group). However, the Spearman test did not reveal a correlation between patients gender and the occurrence of GO, in agreement with data reported by King et al. [9], Margiotta et al. [20], and Güncü et al. [21]. According to Seymour et al. [8], there are evidences that male patients under treatment with nifedipine and cyclosporine are more prone to a greater prevalence and severity of GO than female patients [9, 20]. Since the medication may alter androgen metabolism [22], reaching the gingival fibroblasts, with a consequent increase in the propensity to GO [7, 8]. However, the relation between GO and patient gender acting as a hormonal cofactor has not been completely clarified by this study neither in the literature correlated [22].

In the present study, the dose and the time of nifedipine use were also not correlated with GO [6, 9, 10], in contrast to the data reported by others authors [17, 18, 23]. Probably the severity of GO has not been adequately correlated to pharmacological variables because the events that determine GO depend much more on local factors than on the circulating plasma level of the drug. There are evidences that the drug metabolites concentrated in gingival tissue interact with inflammation chemical mediators, producing a stimuli on fibroblasts activity leading to an unbalance in the local homeostasis, which eventually results in clinically observable GO [24, 25]. Moreover, Seymour et al. [8] reported that the most appropriate method for the assessment of the effect of pharmacological variables is blood analysis, which provides a more precise understanding of the drug dose and GO interaction. 

Concerning the use of the Clinical Index DIGO [11] as a parameter for GO, we noted that the index was easily and rapidly applicable. Considering that most of the indexes for GO assessment are difficult to reproduce, since they require the preparation of casts, photographs, slide projection, and several measurements, this index [11] has proved to be advantageous. The present study seems to be the first one to use Clinical Index DIGO as a method for GO assessment.

The presence and intensity of PI is an important risk factor for the development of GO in patients taking drugs associated with gingival growth [8, 24]. The PI was not correlated with GO in the test group, whereas in the control group, a correlation was observed.

In the control group, GO can be explained by the presence of plaque, which may influence the development of inflammatory gingival overgrowth. These results are in agreement with the literature [5, 19, 23], which could be explained by the fact that the PI for patients with GO were artificially lower due to a possible improvement in oral hygiene before the periodontal examination. 

Gingival inflammation assessed by GI and PB was not significantly different between the test and control groups. However, the correlation test showed association of both GI and PB with the degree of GO severity, and this association was stronger for the test group. This result may explain the influence of these periodontal variables on GO. Gingival inflammation is considered an important risk factor in the expression of GO correlated to nifedipine use [8]. The GI observed in the present study showed results similar to those reported by Barclay et al. [5], King et al. [9], Güncü et al. [21], and Miranda et al. [23]. PB results were similar to those reported by Tavassoli et al. [17], whereas Margiotta et al. [20] did not detect a correlation between PB and GO. 

The PD did not differ significantly between the test and control groups, but GO was associated with an increase in PD in both groups [5, 9, 18, 25]. King et al. [9] assessed CIL in patients under treatment with nifedipine and concluded that this was not a variable correlated with GO. In this study, however, CIL was correlated with GO in the test group for the intervals of mild to severe loss of insertion, which indicates that GO was due to the action of the medication and an association of this effect with periodontal inflammation. The CIL was not correlated with GO for the control group. The *Z* test showed significant differences between the two groups when the loss of insertion was moderate (CIL from 3 to 4 mm). The GO was not defined as significant for the criteria of periodontal variables, which in our study considered the presence of GO to be significant when the score was ≥2. Due to the lack of studies evaluating CIL in patients under treatment with drugs associated with GO, we observed that the assessment of this variable is more indicated in clinical follow-up studies or long-term studies, in which it is possible to evaluate the progress of periodontal disease.

## 5. Conclusion

GO differed significantly between the test and control groups. Its prevalence in the control group may have been due to an inflammatory reaction explained by the influence of periodontal variables (PI, GI, PD, and PB). The prevalence of GO in the test group can be explained by the effect of induction of nifedipine in gingival tissue. The profile of the study group consisted of older people with systemic diseases, mainly cardiovascular diseases, with low socioeconomic status and probably unmotivated with respect to their oral health, which may cause ordinary periodontal inflammation that can exacerbate the gingival overgrowth. The prevalence of GO detected in this study is also related to the use of the Clinical Index DIGO [11] as a method for clinical evaluation, which may be interpreted as a differential factor compared to previous studies. 

## Figures and Tables

**Table 1:** Distribution of the demographic and pharmacological variables in the test and control groups.

	Test group	Control group
Number of patients	35	35
Age (range)	69.5 ± 2.12 (40–80)	40.5 ± 14.85 (30–73)
Gender distribution (m : f)	22 : 13	6 : 29
Nifedipine dose (mg/day)	40 ± 11.21	—
Time of nifedipine use (months)	156 ± 118.79	—

**Table 2:** *Z* test values for the comparison of the proportions for periodontal variables between groups.

Variables	Scores and intervals	*Z* values
		Test × Control

GO	<2	−2.32*
	≥2	2.32*
PI	0-1	−1.22
	2-3	1.24
GI	0-1	0.67
	2-3	−0.79
PD	<3 mm	−1.21
	from 3 to 4 mm	0.57
	≥5 mm	1.19
CIL	<3 mm	−2.68*
	from 3 to 4 mm	2.05*
	≥5 mm	1.31
PB	0	−1.61
	1	1.62

Significant differences: *P < .05*, Z *= ±1.96.

**Table 3:** Spearman correlation among gingival growth (GO ≥ 2), demographic and pharmacological variables for test and control groups.

Variables	Test	Control
	*t value*	*t value*

Age	−1.0383	0.6456
Gender	1.4074	1.4520
Dose	0.3986	—
Time of use	0.5574	—

Spearman Correlation.

Significant difference: *P < .05,* t (tabulated) *= ±2.035.

**Table 4:** Spearman correlation between severity of gingival growth (GO ≥ 2) and periodontal variables for groups.

Periodontal variables		Test * t value *	Control *t value *
PI	(2-3)	1.5366	2.6269*
GI	(2-3)	2.9879*	2.5203*
PD	(<3 mm)	−5.4483*	−2.9233*
	(from 3 to 4 mm)	4.4462*	2.6914*
	(≥5 mm)	5.4593*	2.1133*
CIL	(<3 mm)	−2.2788*	−1.9358
	(from 3 to 4 mm)	0.6250	1.9852
	(≥5 mm)	4.6975*	1.9970
PB		3.5932*	2.5119*

Spearman Correlation.

Significant difference: *P < .05,* t (tabulated) *= ±2.035.
